# Epigenetic signatures of migratory orientation in a songbird hybrid zone

**DOI:** 10.1098/rstb.2025.0021

**Published:** 2026-07-23

**Authors:** Oluwamosope Adeniji, Hannah Justen, Bernice Sepers, Alan Pepper, Kira Delmore

**Affiliations:** 1 Department of Biology, Texas A&M University, College Station, TX 77843, USA; 2 Neurobiology, Physiology and Behavior Department, University of California, Davis, CA 95616, USA; 3 Department of Evolutionary Population Genetics, Bielefeld University, Bielefeld, D-33501 Nordrhein-Westfalen, Germany; 4 Columbia University, New York, NY 10027, USA

**Keywords:** songbird, DNA methylation, migration, speciation, hybrid, behaviour, evolution, genomics

## Abstract

Epigenetic marks are strong candidates for elements controlling environmentally mediated behaviours like migration. Understanding the potential role of epigenetics in migratory behaviour is increasingly important, as rapidly changing environmental conditions threaten many migratory populations. Here, we report a methylome-wide analysis to identify differentially methylated CpG sites (DMSs) associated with migratory orientation in the Swainson’s thrush (*Catharus ustulatus*), a migratory songbird with two subspecies that show distinct migratory pathways. We compared birds with extreme autumn migration orientations, including pure forms and early-generation hybrids, and contrasted our epigenomic results with genomic variation. We found weak methylome-wide differentiation, largely explained by ancestry and heterozygosity, but identified a small number of DMSs based on migratory orientation. These DMSs were located within transposable elements, while others were evenly distributed between promoters and gene bodies. Genes with annotated roles in olfaction were enriched near these DMSs, supporting growing evidence for their role in migratory orientation. Many DMSs were also linked to genetic variants in *trans*-regulatory regions, suggesting that methylated sites may interact with distant genetic variants to influence migratory orientation in response to environmental cues. These findings advance our understanding of the molecular mechanisms that control differences in migratory orientation, providing empirical evidence of epigenetic divergence with potential implications for intraspecific divergence in migratory behaviour.

This article is part of the theme issue ‘Ecological epigenetics at the intersection of behaviour and life history variation in non-model animals’.

## 1. Introduction

Seasonal migration—the annual movement of individuals between their breeding and non-breeding grounds—is a complex behaviour that requires the integration of multiple traits, including morphological, life history, sensory, physiological and behavioural traits [1]. Many of these traits vary both within and between populations (e.g. populations can differ in when they arrive on the breeding grounds, where they winter and the aerodynamic features of their wings) [2, 3]. One active area of research in the field of migration involves identifying molecular mechanisms that underlie variation in migratory traits [4, 5]. This area of research is becoming increasingly urgent, as rapidly changing environmental conditions are threatening many migratory populations and may require changes in their migratory behaviour [6, 7].

Most of the research on mechanisms underlying migratory traits has focused on genetic variants. Initial work began with analyses of candidate genes, surveying variation at genes with functions that could be related to migration (e.g. variation in the size of the polyQ domain of the *Clock* gene [8, 9]). Recently, work on the genetics of migration has moved towards unbiased approaches using next-generation sequencing techniques. Variation at several SNPs (single nucleotide polymorphisms; e.g. [10, 11]) and structural variants (e.g. [3, 12, 13]) has been connected to migratory traits using these techniques, but variation at these markers and candidate genes cannot account for the full extent of phenotypic variation present in migratory traits [3, 14].

Work on epigenetic variation underlying migratory traits may help gain a more complete understanding of the mechanisms underlying this complex behaviour. Epigenetic variation can include alterations to chromatin structure, the activity of transcription factors that bind *cis*-regulatory regions and/or the methylation status of DNA [15]. Epigenetic markers can act independently of genetic variation and are known to play a role in mediating the relationship between an individual’s genome and the environment [16, 17]. They may be stably inherited across multiple generations or dynamically adjusted in response to environmental factors [18–21]. This connection with the environment makes epigenetic variation a particularly strong candidate for controlling migration, because many migratory traits are tightly connected with environmental cues. For example, birds use the sun and polarized light [22, 23], the earth’s magnetic field [24, 25] and the stars [26] to orient during migration.

There is relatively little work on the epigenetics of migration that has focused primarily on DNA methylation. This epigenetic modification alters gene expression by adding a methyl group—often to CpG dinucleotides on the fifth carbon of the cytosine base. When this addition occurs in promoter regions, it can influence promoter usage, modulate enhancer activity and lead to downregulation of the associated gene [27, 28]. In the context of migration, differentially methylated CpG regions (DMRs) have been shown to distinguish migratory from non-migratory individuals in salmonids. These DMRs are located in regulatory regions of genes implicated in key migratory functions, including circadian rhythm regulation, energy metabolism and muscle performance [29]. Similar patterns have been observed in birds. For example, Saino *et al*. [30] found that the increased CpG methylation at the poly-Q and 5 ′ -UTR loci of the photoperiodic *Clock* gene in barn swallows was associated with earlier spring departures, earlier arrival at breeding sites and earlier breeding dates. Note, Saino *et al*. [30] examined patterns of methylation in the blood. This is often the target tissue in avian studies, where terminal sampling may be difficult. Tissue-specific epigenetic patterns may be missed when using blood, but positive correlations between DNA methylation patterns in the blood and other tissues (e.g. brain and liver) have been documented [21, 31], suggesting that blood may be a suitable proxy tissue when terminal sampling is not possible.

**Figure 1.**
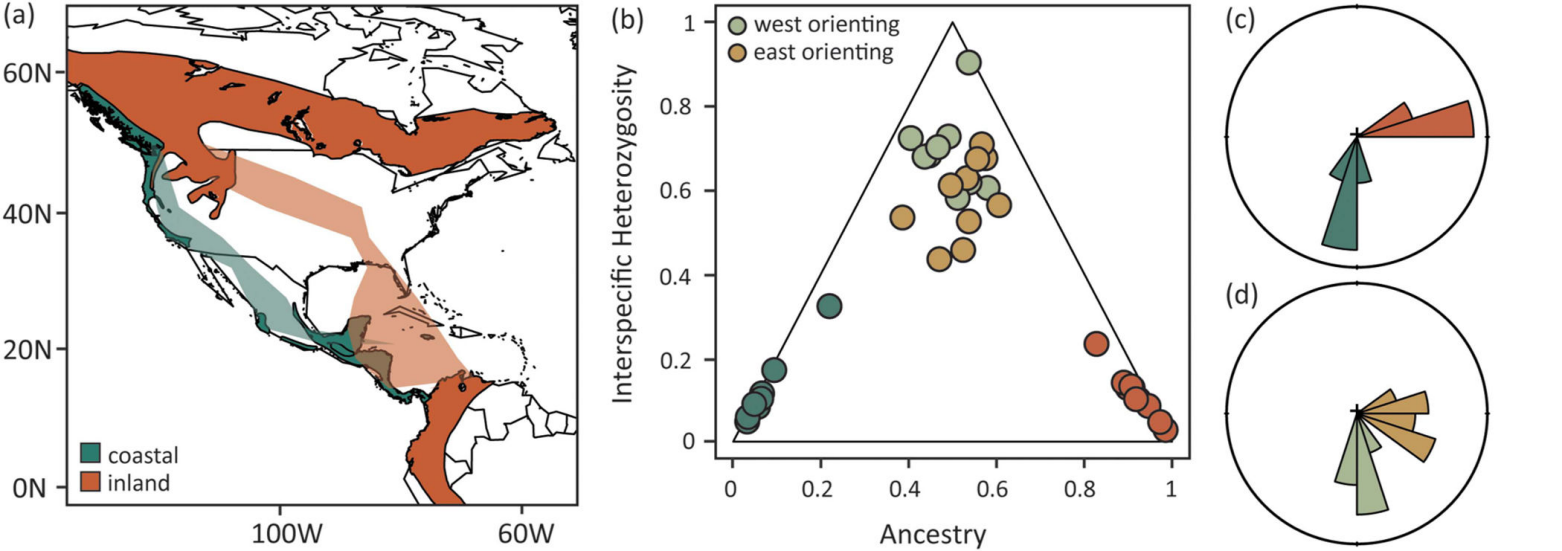
Study system and sampling design. (a) Breeding and wintering distribution of coastal (green) and inland (orange) thrushes. Polygons summarize migration tracking data from pure forms tracked during migration in [32]. (b) Genomic ancestry and interspecific heterozygosity estimated for juvenile male birds in the study, including 18 pure forms with extreme orientations (*n* = 10 inland; 8 coastal) and 20 early-generation hybrids with extreme orientations (*n* = 10, east [mint green]; 10 = west [gold]). Circular plots indicate the initial autumn orientation of (c) pure forms and (d) early-generation hybrids.

In the present study, we will expand work on the epigenetics of migration using the Swainson’s thrush (*Catharus ustulatus*). The Swainson’s thrush includes two subspecies—inland and coastal (*C. u. swainsoni* and *C. u. ustulatus*, respectively)—that form a well-characterized migratory divide. Specifically, these two subspecies hybridize where their breeding ranges overlap in western North America but follow distinct migratory routes. The coastal subspecies migrates south along the Pacific Coast to Mexico, Guatemala and Honduras, while the inland subspecies migrates southeast across the Rocky Mountains through central North America to Colombia and Venezuela (figure 1) [32]. Variation in several migratory traits exists in this system, including not only orientation but also the distance, timing of migration and wing morphology [3, 33]. Although genetic variants underlying some of these traits have been identified, they do not fully explain the observed phenotypic variation. For example, genomic heritability estimates for orientation range from 0.30 to 0.55 [3]. Many additional variables probably help explain variation in the migratory behaviour of thrushes (e.g. developmental plasticity, parental effects, ecological and/or cultural transmission) [34–36]. In the present study, we tested the effects of epigenetic variation.

We focus specifically on DNA methylation and use radio tracking data from a hybrid population of thrushes to unravel the epigenetic basis of migratory orientation. The hybrid zone between thrushes includes birds categorized on the basis of genotype as pure coastal or inland, early- and later-generation hybrids and backcrosses [37–39]. Birds genotyped as pure orient in directions that match those of their respective allopatric populations (i.e. south [coastal] or southeast [inland]); hybrids exhibit a wide range of orientations, from directly south through to southeast orientations, including orientations intermediate between these two directions [3, 33]. We included both pure forms and early-generation hybrids in the present analysis, focusing on birds with extreme orientations (figure 1). Our first objective was to test for methylome-wide differentiation that could be related to ancestry, interspecific heterozygosity and/or migratory orientation. We contrasted results from this analysis with genome-wide patterns of differentiation derived from whole-genome resequencing data generated for the same set of birds. Our second objective was to identify specific differentially methylated CpG sites (DMSs) related to migratory orientation, looking for DMSs that distinguished both pure forms and hybrids with extreme orientations. DMSs that distinguish pure forms could derive from any number of factors (e.g. genetic variants that distinguish the subspecies and affect epigenetic markers that are not associated with migration); DMSs that also distinguish early-generation hybrids with extreme orientations should be more specific to orientation. In our third and final objective, we tested for associations between genetic variants (SNPs) and variation in DNA methylation. We distinguished between SNPs in *cis* (local) and *trans* (distant) regulatory regions in this last objective. This knowledge is important for understanding molecular and evolutionary mechanisms underlying phenotypic change. For example, localized *cis*-regulatory changes are thought to have fewer pleiotropic effects, allowing them to evolve with fewer constraints, while more global *trans*-regulatory changes are thought to respond more readily to environmental cues [40].

## 2. Methods

Birds in the present study were sampled and tagged under protocols approved by the Institutional Animal Care and Use Committee at Texas A&M University (IACUC Protocol #2024–0008) and Environment and Climate Change Canada (10921).

### (a) Phenotypic data collection

We focused our analysis on one migratory trait: the initial outward orientation of birds away from the breeding grounds during autumn migration (hereafter, ‘autumn orientation’) and males. We focused on juvenile male birds only to control for age and sex effects. Detailed methods for quantifying migratory orientation are described in [3, 38]. Briefly, we fitted birds with radio tags in Pemberton, British Columbia, between August and September 2019–2022. These radio tags are set to be picked up by the Motus Wildlife Tracking System (motus.org [41]). Motus is an autonomous network of radio towers that includes both preexisting receiver stations found globally and our own fence of stations that spans the width of the hybrid zone between Swainson’s thrush subspecies. Birds fitted with radio tags are picked up as they pass the preexisting stations and our fence. To estimate autumn orientation, we defined a 300 km-diameter circle around our release site and calculated the bearing from the release site to the first detection location within this circle using the geosphere package in R [42].

We have tracked *>* 600 birds on migration using this approach [38]. In the present study, we limited our analysis to juvenile males and selected 18 individuals that were designated as genetically pure (8 coastal and 10 inland) and 20 individuals designated as early-generation hybrids that exhibited extreme latitudinal orientations during autumn migration (10 from each extreme; figure 1; supplementary material, table S1). Details on how birds were designated as genetically pure, or early-generation hybrids, are provided in the next section.

### (b) Tissue sampling, whole-genome resequencing and analysis

We collected blood samples at the time of bird tagging. Approximately 50 μl of blood were collected from the brachial vein and stored in Queens Lysis Buffer. Genomic DNA was extracted from these samples using a standard phenol–chloroform protocol and used to prepare both whole-genome resequencing libraries and whole-genome methylation profiles. Methods for whole-genome resequencing can be found in Justen *et al*. [3], where these data were initially presented. Briefly, we prepared libraries following [43] and [44] and sequenced these libraries to low coverage (average read depth 3.8 ×) on an Illumina NovaSeq 6000. We trimmed the resulting sequences and aligned them to reference genomes assembled for both the inland and coastal Swainson’s thrushes [45, 46]. We limited subsequent steps to reads that mapped uniquely to both references and their mapping positions in the inland reference. The resulting BAMs were used to generate two sets of genotypes: (i) ancestry informative markers (AIMs) and (ii) SNPs.

Methods for calling AIMs can be found in Blain *et al*. [38]. Briefly, a set of loci that distinguish inland and coastal thrushes was identified using their reference genomes and birds from allopatric populations sequenced to high coverage (10–15 ×). A hidden Markov model was then used to infer ancestry for each individual and AIM, given reference species allele counts and the genetic distance between loci. We filtered this dataset to include AIMs with minor allele frequencies *>* 5%, missing data *<* 25% and only autosomal loci (*n* = 1517 AIMs). This filtered set of AIMs was used to estimate ancestry and interspecific heterozygosity, allowing us to identify pure and early-generation hybrids. Genome-wide ancestry was calculated as the mean AIM value across all loci; interspecific heterozygosity was calculated as the proportion of heterozygous sites per individual (i.e. sites where birds had alleles diagnostic of both the inland and coastal subspecies). Birds with ancestry ≤ 0.2 or ≥ 0.8 were considered pure coastal or inland, respectively. For hybrids, birds with ancestry between 0.4 and 0.6 and heterozygosity exceeding 0.5 were considered early generation.

SNPs were used for all other genomic analyses (i.e. for all analyses beyond placing birds into different ancestry categories, including redundancy analysis (RDA) and association analyses; see details below). Methods for calling SNPs can be found in Justen *et al*. [3]. Briefly, we used STITCH (Sequencing To Imputation Through Constructing Haplotypes [47]) to generate this dataset. STITCH uses low-coverage sequencing data to model ancestral haplotypes and a hidden Markov model to estimate genotype probabilities for each individual. We filtered the resulting set of SNPs to those with allele frequency *<* 5%, missing data *>* 25% and those failing linkage pruning (parameters: 200 20 0.2; *n* = 170 771 SNPs).

### (c) Whole-genome methylation sequencing

Whole-genome methylation profiles were generated for the present study using the same genomic DNA as used for whole-genome resequencing. We checked the genomic DNA for purity using a NanoDrop and quantified it using a Qubit High Sensitivity Assay Kit (Thermo Fisher Scientific). We sheared 100–200 ng of this DNA to 240–290 bp on a Covaris S2 (settings: 1 × 90 seconds followed by 2 × 45 seconds, yielding a 300 bp target size) and used it as a template for libraries prepared with the NEBNext Enzymatic Methyl-seq Kit (EM-seq; New England Biolabs). We spiked the libraries with DNA from two controls, CpG-methylated pUC19 (positive control) and unmethylated lambda (negative control). These controls were used to quantify the conversion efficiency of methylated cytosine in the resulting libraries. We modified the manufacturer’s protocols slightly to reduce target DNA loss and improve methylated cytosine conversion efficiency. Specifically, during the purification steps, we gently mixed magnetic beads with the suspended DNA fragments by pipetting up and down 20 times. We extended the incubation time at room temperature on the benchtop to 10 minutes to ensure optimal binding of DNA fragments to the beads. To avoid over-drying, we air-dried the beads for only 30–60 seconds, keeping them slightly wet—especially during the clean-up of deaminated DNA. We eluted the DNA by slowly pipetting the elution buffer up and down at least 20 times to maximize recovery. We also increased the separation time on the magnetic stand to 5 minutes to ensure complete separation of the DNA from the beads. For PCR amplification, we used a maximum of six to eight cycles, depending on the initial quantity of sheared DNA as measured by Qubit, to minimize PCR duplicates. We quantified the final libraries using both the Agilent D1000HS TapeStation and the Qubit dsDNA High Sensitivity Assay Kit. Libraries were sequenced on an Illumina NovaSeqX Plus with a 15% PhiX spike-in to increase nucleotide diversity. Note, each hybrid library was sequenced twice, with the average read depth for the first set of libraries being 4.54 × (range 1.64 × to 13.57 ×) and the average read depth for the second set being 8.16 × with a range of 6.75 × to 13.57 ×. Finally, the pair of libraries for each individual was merged using SAMtools v1.20 to reach the final average read depth of 9.61.

### (d) EM-Seq data analysis

#### (i) Alignment

Raw FASTQ files containing sequencing reads and trimmed reads were quality checked using FastQC v0.11.9 (https://www.bioinformatics.babraham.ac.uk/projects/fastqc/) and summarized with MultiQC v1.14 [48]. Trim Galore v0.6.7 and Cutadapt v1.18 (https://github.com/FelixKrueger/TrimGalore) were used to remove low-quality sequences (Quality Phred score *<* 20) and artificial adaptor sequences using the following parameters: ’–clip_R1 10 –clip_R2 10 –three_prime_clip_R1 10 – three_prime_clip_R2 10’. The trimmed read-pairs were then aligned in non-directional mode to bisulphite-converted versions of reference genomes available for both the inland and coastal subspecies of thrush using Bowtie 2 within Bismark v0.24.0 [49]. Using Bismark, the resulting Binary Alignment Map (BAM) files were deduplicated and filtered to retain only uniquely mapped reads. To remove alignment bias to either reference genome, we used *ngsutilsj* (https://github.com/compgen-io/ngsutilsj) to identify reads that mapped to both genomes; all downstream analyses were restricted to these reads, including final alignment to the inland reference genome, chosen for its higher contiguity and completeness (e.g. BUSCO score = 96.8% vs. 94.2% for the coastal genome).

#### (ii) Methylation calling and filtering

Cytosine methylation was called by generating a cytosine report file using Bismark’s v0.24.0 ‘bismark_methylation_extractor’ with the following options: ‘–cytosine_report –CX –gzip –cutoff 2 –bedGraph –comprehensive –counts –report –merge_non_CpG –multicore 8’. To remove potential confounding from C/T and G/A SNPs, we generated a mask using SNPs from Justen *et al*. [3], who generated low-coverage whole-genome resequencing data from 600 hybrid thrushes. SNPs were filtered using *bcftools* (‘–min-BQ 20 –min-MQ 20 –QUAL *>* 500 –skip-variants indels’ [50]) and retained if they had a minor allele frequency *>* 5% and a missing genotype frequency *<* 25%. SNPs with C/T or G/A alleles were extracted and merged using BEDOPS v2.4.35 [51] to generate a Browser Extensible Data (BED) file for masking. Finally, the conversion efficiency was assessed by aligning reads to the lambda and pUC19 control sequences. We focused on CpG methylation to identify epigenetic differences within and between bird groups. All downstream analyses were conducted in R v4.3.1 [52], which includes an adaptation of the FilteringPlotting.R script from https://github.com/rshuhuachen/great-tit-methylation-genes-environment.

We examined global CpG methylation patterns by calculating cytosine percentage methylation in CG contexts. A one-way ANOVA was used to test differences in average methylation levels between pure and hybrid individuals. We then filtered CpG site methylation data using methylKit v1.16.1 [53]. Reads with coverage under *<* 8 × and above the 99.9th percentile were excluded to minimize PCR bias. Coverage values were normalized across samples using a median-based scaling factor. When de-stranding filtered data (i.e. combined the readings of both strands of a CpG site), we limited our analysis to CpGs shared by more than 60% of the total number of birds for each category within pure forms or hybrid group. Furthermore, we removed CpGs that were either totally methylated or completely unmethylated in all individuals.

#### (iii) Redundancy analyses

To check whether birds clustered by ancestry, heterozygosity and/or autumn migratory orientation, we used vegan v2.6–8 [54] to perform a RDA using the filtered CpG methylation dataset, and further limiting the analysis to CpG sites with *<* 5 missing values for all birds (38 in total) and imputing with the mean value of the respective CpG sites for the remaining missing sites or cross-individual mean values of the corresponding CpGs. Using CpG sites as the dependent variable and ancestry, heterozygosity and autumn orientation as explanatory variables in our RDA model, we calculated the overall RDA *p*-value using 5000 permutations. *p*-values for each RDA axis were estimated using 2000 permutations each. For comparison, we conducted complementary RDAs with the filtered SNPs (genotype probabilities) described above, generated from whole-genome resequencing data.

#### (iv) Methylation differentiation

We used the callDML function from the DSS package v2.50.1 [55] to identify differentially methylated CpG dinucleotides. We used beta-binomial regression with an arcsine link function to estimate test statistics, considering sites to be differentially methylated by applying a significance threshold of *p <* 0.05 and a minimum methylation difference (delta) of 0.05. To correct for multiple testing, we employed a false discovery rate (FDR) [56]. Sites below the 0.1 FDR threshold were retained for downstream analysis. We report DMSs from three comparisons: (i) pure coastal versus pure inland, (ii) westward- versus eastward-orienting early-generation hybrids, and (iii) combining data by phenotype regardless of ancestry (i.e. comparing pure coastal and westward-orienting hybrids with pure inland and eastward-orienting hybrids). Our reasoning for the last comparison is that one of our objectives was to identify loci that overlapped between both pure forms and extreme hybrids (comparisons 1 and 2), assuming that these loci were more likely to be related to orientation. We found limited overlap when we compared DMSs between pure forms and extreme hybrids. A lack of power could be responsible for this limited overlap and could be improved by combining data by orientation phenotype. We limited our subsequent analyses (genomic feature annotation and Gene Ontology enrichment analysis) to loci that overlapped between our two initial comparisons (between pure forms and extreme hybrids) and loci that overlapped between comparisons 1 and 3 (between pure forms and when combined). Similarly, DMRs were identified using the DSS callDMR() function and applying a significance threshold of *p <* 0.05.

#### (v) Genome feature annotation and Gene Ontology enrichment

Significant DMSs and DMRs were annotated using ‘bedtools intersect’ and ‘bedtools closest’ from BEDTools v2.30.0 [57], with a curated set of genomic features from the inland Swainson’s thrush reference genome (GCF_009819885.1), which includes 19 270 predicted protein-encoding genes. Genomic features were extracted and classified using Bioconductor packages including GenomicRanges v1.54.1, rtracklayer v1.62.0 and GenomicFeatures v1.54.4. Promoters were defined as regions spanning 2000 bp upstream and 50 bp downstream of transcription start sites. Exons, 5 ′ UTRs and 3 ′ UTRs were extracted based on gene or transcript annotations. Introns were identified using ‘intronsByTranscript’ from GenomicFeatures and removing overlaps with any other annotated genome feature. Intergenic regions were defined as intervals between annotated genes. CpG islands (CGIs) were imported from UCSC Genome Browser GTF files. Transposable elements (TEs) were obtained from a curated GFF2 file specific to *Catharus* (the genus to which the Swainson’s thrush belongs) and as described in [58].

Genome Ontology (GO) enrichment for genes that overlap or are less than 25 kb distant from a DMS was examined using OmicsBox v3.4.6 and REVIGO (http://revigo.irb.hr/). *p*-values were corrected using the Benjamini–Hochberg FDR method (FDR *<* 0.05). Only GO terms that included at least two genes and had a significance level *p*-value *<* 0.05 were considered for further analysis. We used REVIGO to remove redundant and overlapping GO categories, with the default semantic similarity measure.

#### (vi) Association analysis of methylation and genetic variants

To identify genetic variants associated with variation in DNA methylation, we performed an association analysis using the R package Matrix eQTL v2.3 [59] and our SNP dataset. In this analysis, we tested for genome-wide association between CpG sites and SNPs using the DNA methylation level at each CpG site as the phenotype. We used matrixeqtl.R and filter_matrixeqtl.py scripts with some modifications, available at https://github.com/rshuhuachen/great-tit-methylation-genes-environment. This analysis was conducted to detect significant associations between AIMs and DMSs across all individuals, grouped by shared autumn migratory orientation (east vs. west). We tested each CpG–SNP pair independently for associations by modelling the effect of SNP genotype (the SNP encoded by 0, 1 and 2) on methylation levels at all DMSs used in previous RDA and including covariate as autumnl orientation, eastward or westward. CpG–SNP pairs were tested for *cis*-associations (*<* 1 Mbp distance) and *trans*-associations (*>* 5 Mbp distance). FDR correction was applied separately for *cis-* and *trans-* tests, and associations with FDR-adjusted *p*-values *<* 0.05 were considered statistically significant.

## 3. Results

### (a) Global methylation patterns

The average read depth for all 38 samples was 8.64 ×, and alignment scores ranged between 74.8% and 85.9%, with an average of 80.1%. To assess the enzymatic conversion efficiency of methylated cytosines throughout the genome, we evaluated methylation levels in control DNA. The positive control showed 84.2%–98.6% CpG methylation and 0.2%–1.7% in non-CpG context. For negative control, percentage methylation ranged between 0.1% and 0.7% in both contexts, confirming efficient conversion. Cytosine percentage methylation in CG contexts ranged between 42% and 50%, consistent with previously reported levels in avian genomes [60, 61]. No significant differences in overall CpG methylation percentage were observed across the four groups (*F* = 1.03, *p* = 0.391; supplementary material, figure S1). We retained 27 226 potentially informative CpG sites per individual for downstream analysis after data filtering.

### (b) Variation at the whole methylome and genome level

**Figure 2.**
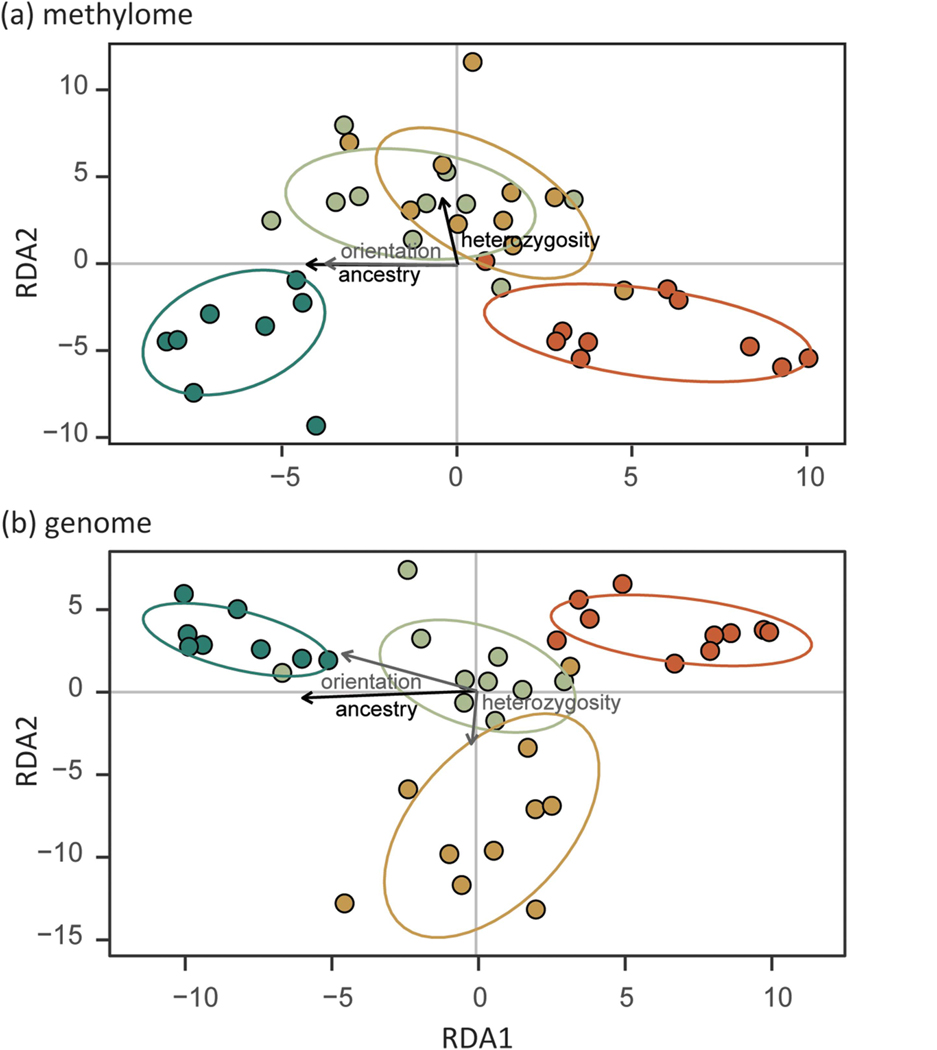
Redundancy analyses using (a) methylation and (b) genomic data. Each point represents an individual bird, with coastal in green, hybrids with westward orientation in mint-green, hybrids with eastward orientation in gold and inland in orange. Ellipses indicate confidence intervals around each group. Arrows represent explanatory variables, with long arrows indicating large amount of variance explained by the variable, and black colour showing those that are significant (*p <* 0.05).

Out of the 27 226 CpG sites, an RDA of 13 639 methylated CpG sites using autumn orientation, genome-wide ancestry and interspecific heterozygosity as explanatory variables was significant (*p*-value = 0.001) but explained only a small proportion of the variance (*R*^2^ = 0.019). RDA1 axis explained a significant amount of variance in the methylated data (RDA1 = 4.44%, *p*-value = 0.003) and RDA2 explained a non-significant amount of variance (RDA2 = 3.41%, *p*-value = 0.21). Two variables were significant predictors (ancestry: *p*-value = 0.0004; heterozygosity: *p*-value = 0.047). Ancestry and autumn orientation were largely represented by RDA1; heterozygosity by RDA2 (figure 2a).

RDA using SNPs (170 771 SNPs) and the same set of explanatory variables was also significant (*p*-value = 0.001) and explained a larger proportion of the variance (*R*^2^ = 0.06) than methylome data (figure 2b). RDA1 explained a significant amount of variance in the genomic data (8.8%, *p*-value = 0.00050) but RDA2 did not (3.17%, *p*-value = 0.21). One variable was a significant predictor (ancestry: *p*-value = 0.0002). Ancestry and autumn orientation were largely represented by RDA1; heterozygosity by RDA2 (figure 2b).

### (c) Differentiation at the local methylation level

**Figure 3.**
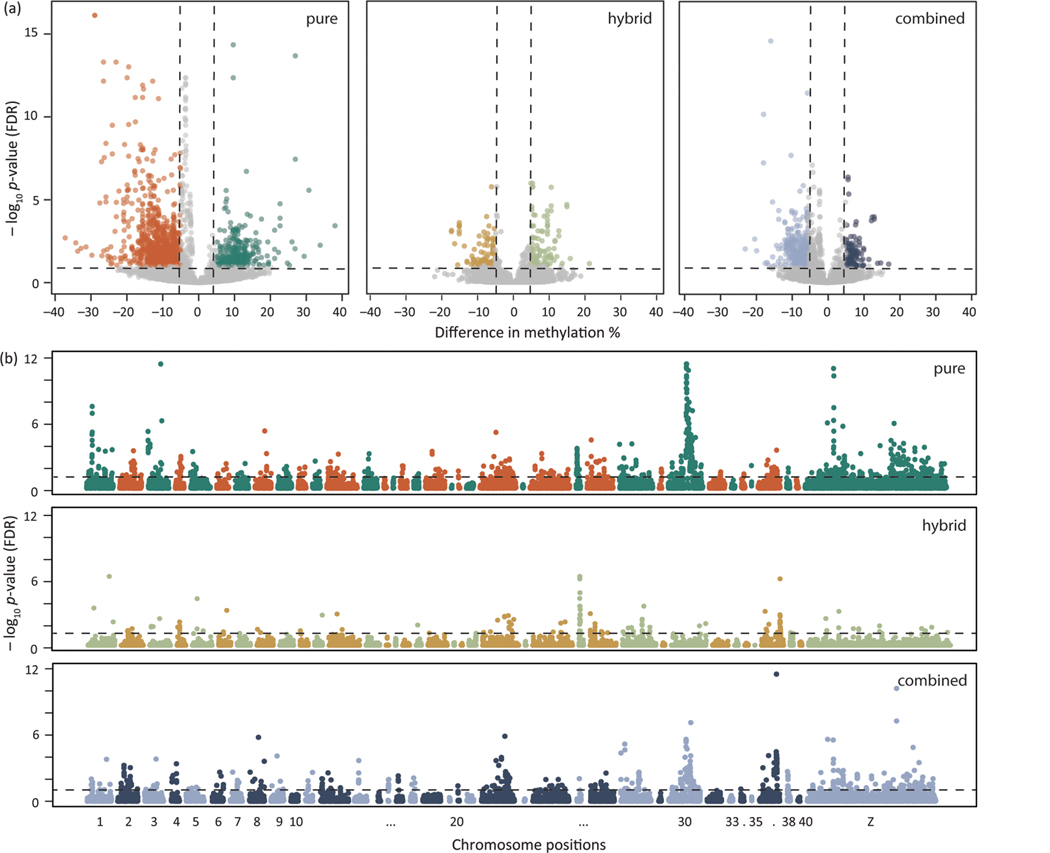
Local patterns of differential methylation. (a) Volcano plots. Pairwise comparison (from left to right) of DMSs: between pure forms (relative to coastal birds), between hybrids (relative to westward-oriented hybrids) and combined birds (relative to all westward-oriented birds). Vertical dashed lines indicate FDR = 0.05, and horizontal dashed lines indicate a 5% difference in methylation level. Grey dots represent DMSs below the cutoff for significance thresholds and differences in methylation levels. Dots on the left, including red, gold and light-blue colours, indicate a significant loss in methylation level and those on the right, including green, mint-green and dark blue, indicate a significant gain in methylation level. (b) Manhattan plots showing the location of DMSs along the genome. Horizontal dashed lines mark the genome-wide significance threshold of the FDR-adjusted *p*-value (*p <* 0.05).

We tested for differential methylation using the 27 226 potentially informative CpG sites and started with comparisons of pure coastal versus pure inland birds and westward- versus eastward-orienting hybrids. About 1068 CpG sites were differentially methylated between pure forms (*p <* 0.05, *Δ*= 0.05); 784 remained significant after FDR correction and were found on most chromosomes, with a distinct cluster on chromosome 30 (figure 3). In assessing the genome regions where DMSs were concentrated, 47 significant DMRs (*p <* 0.05) were identified, each containing at least 4 CpG sites (maximum 10), ranging from 66 to 3238 bp in length. Moving on to hybrids, overall levels of differential methylation were lower. Eighty-one DMSs were identified and reduced to 77 significant sites after FDR correction (figure 3). There were 4 significant DMRs containing at least 4 CpG sites (maximum 12), ranging in length from 79 to 278 bp.

**Figure 4.**
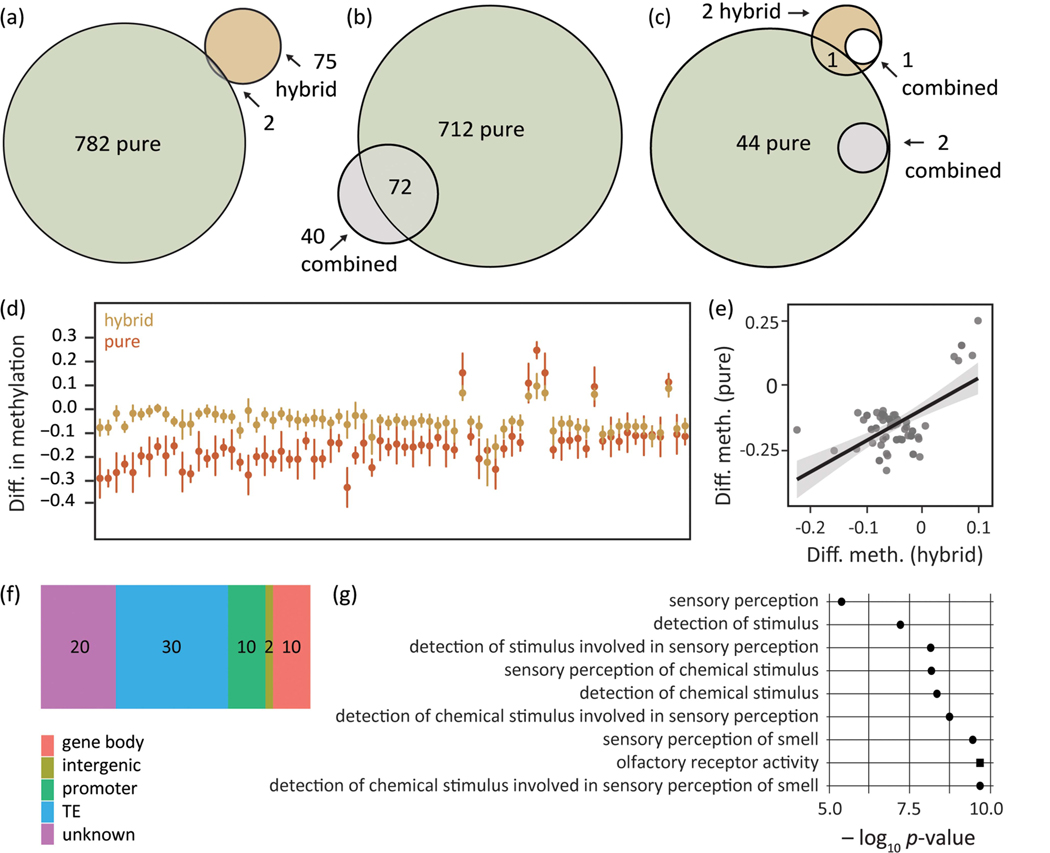
Details on DMSs and DMRs. (a–c) Venn diagrams showing overlap between groups in (a,b) DMSs and (c) DMRs. Overlap for pure versus hybrid and pure versus combined is shown together in (c). (d,e) Comparing differences in methylation estimated at the 72 DMSs that showed consistent patterns in the pure and combined comparisons for pure forms (orange) and hybrids (gold). Values of differential methylation are plotted in (d), consistent but less extreme values in hybrid comparisons, and the correlation between these values in (e). (f) Genomic features the 72 DMSs overlap with (block width reflects the number of DMSs that occurred in each feature). (g) Results from GO analysis showing 9 overrepresented GO terms from 26 genes. Shapes indicate the general classification of GO terms, with circles representing biological processes and squares for molecular function.

We were specifically interested in DMSs that are found in both comparisons (i.e. both in pure coastal versus pure inland birds and in westward- versus eastward-orienting hybrids) because there is a higher chance that they are important for orientation. Only two DMSs (figure 4a) and one DMR (figure 4c) overlapped between these comparisons. This small number could be the result of low statistical power, as we only had 8–10 individuals per group. Consequently, we ran a third analysis in which we grouped birds by phenotype (autumn orientation) regardless of ancestry (i.e. compared pure coastal and westward-orienting hybrids with pure inland and eastward-orienting hybrids, *n* = 18–20 per group in this case). After FDR correction, 112 significant DMSs and 3 DMRs were identified, each with at least 4 CpG sites (maximum 7), ranging from 89 to 104 bp in length (figure 3).

Seventy-two of the 112 DMSs in the combined analysis were also found in our comparison of pure forms (figure 4b) and 2 DMRs (figure 4c). As shown in figure 4d, the direction of differential methylation at all 72 of these sites is the same when only hybrids are examined, but differences in methylation did not exceed our threshold for being considered differentially methylated (*Δ*= 0.05), explaining why they were not recovered in the hybrid-only analysis. In support of this observation, there was a strong, positive and significant relationship between values estimated in the pure and hybrid-only analyses (Pearson’s correlation, *R*^2^ = 0.59, *p <* 0.0001; figure 4e). Note, the 2 DMSs that overlapped between pure forms and hybrid groups were also in the list of 72 DMSs that overlapped between pure forms and our combined comparison.

### (d) Genome features and gene enrichment analysis

Genome enrichment analysis was performed on the 72 DMSs that were identified in both the analyses of pure individuals and birds combined by migratory orientation, regardless of ancestry. Fifty-two DMS overlapped with known genome features; the remaining were found on scaffolds that have not been assigned to chromosomes and have no annotated features. Many of these 52 DMSs were in TEs, and there was an equal representation in promoters and gene bodies. Intergenic regions were the least represented (figure 4f). These 52 DMS occurred in promoters or gene bodies of 33 genes. Twenty-six of these genes had associated GO terms. A GO analysis using these genes found enrichment of nine GO terms (FDR *<* 0.05; figure 4g; supplementary material, table S2).

### (e) Association analysis

A total of 722 023 CpG–SNP pairs were tested for *cis*-associations with SNPs (*<* 1 Mbp distance), and 2 328 423 646 pairs were tested for *trans*-associations (*>* 5 Mbp distance). Fifty-two *cis*-associations were significant after FDR correction for multiple testing (FDR *<* 0.05). We identified 85 872 significant *trans*-associations between DMSs and SNPs on different chromosomes (FDR *<* 0.05). Among the 72 significant DMSs found in both the combined and pure form comparisons, there was zero overlap with *cis*-associations and 14 unique overlaps with significant *trans*-associations. These 14 overlaps were in 2 uncharacterized and 3 functional genes, including TOG array regulator of axonemal microtubules protein 2, olfactory receptor 14J1 and H/ACA ribonucleoprotein complex subunit 3.

## 4. Discussion

We used radio tracking, data on DNA methylation and a hybrid zone between two subspecies of Swainson’s thrushes to provide one of the first methylome-wide studies of a specific migratory trait that markedly differs between the two subspecies, autumn orientation. We found weak methylome-wide differentiation, but a subset of differentially methylated variants did distinguish birds with different orientations and may help control this behaviour. These variants occurred in genes that are important in sensory reception, especially olfaction, and (when linked to genetic variants) variation in *trans*-regulatory regions dominated.

We documented only weak methylome-wide differentiation. This variation was explained by ancestry and heterozygosity but not autumn orientation, suggesting that differences in methylation relate more to subspecies-level differences than migratory orientation. Hence, differences in migratory orientation do not generate strong methylome-wide patterns of differentiation in the Swainson’s thrush. Future work with larger sample sizes and additional tissues beyond blood is needed to validate this suggestion, but combined with our local methylome analyses (see below), this finding could suggest that the connection between differential methylation and migratory orientation is limited to a small number of regions in the methylome. We are not aware of any comparable methylome-wide work on migratory behaviour, but other work in songbirds has documented strong relationships between methylation at candidate genes and migratory traits. Specifically, in female barn swallows, methylation at *Clock* poly-Q respectively explained 50% and 42% of the variation in departure date from the wintering ground and arrival date to the breeding grounds [30]. Values were lower for male barn swallows (the sex that we focused on here; 3% and 7%, respectively) and without methylome-wide data these authors cannot rule out a role for other (perhaps methylome-wide) effects of migration on the methylome. Justen *et al*. [3] also identified a small number of genomic (vs. methylomic) regions underlying migratory orientation in thrushes. Future work in thrushes could assay other epigenetic markers that could underlie variation in migration (e.g. chromatin accessibility).

Although we did not document a methylome-wide association between orientation and differential methylation, we did identify a small number of differentially methylated sites and regions linked to orientation in our local analysis. Of the DMSs linked to functional genes, some were enriched for olfactory receptor activity and detection of chemical stimulus involved in the sensory perception of smell. Interestingly, a phylogenomic study of *Catharus* (the genus that includes Swainson’s thrushes) found enrichment of similar GO terms in windows that grouped migratory (vs. resident) *Catharus* species, including olfactory receptor activity and G protein-coupled receptor activity [62]. Birds rely on multiple sensory cues during migration, including olfactory signals. Olfactory cues, such as volatile chemicals detected by receptor proteins, are important for intermediate-scale navigation, and pigeons have demonstrated the ability to form odour-based navigational maps by associating wind-borne odours with wind directions at their home location [63, 64]. Although birds are generally thought to rely less on olfaction than mammals, their genomes contain many olfactory receptor genes (approx. 640), with approximately 53% being functional [65]. Concerning the specific role that olfactory receptors play in thrushes, coastal and inland subspecies occur in different forest types (riparian woodlands vs. boreal forest). Perhaps coastal birds have increased expression of receptors sensitive to the riparian woodlands that characterize the Pacific Coast of North America, directing them towards flyways along the Pacific Coast. Conversely, decreased activity in certain olfactory receptor pathways may shift dependence to alternative mechanisms important for orientation, including magnetoreception, vision and even acoustic cues [64]. Note, we measured levels of methylation in the blood. Accordingly, the differential methylation of olfactory receptor genes that we observed in blood may reflect global changes in olfactory receptor gene methylation that have functional consequences in other tissues—such as olfactory neurons. In the great tit (*Parus major*), temporal changes in methylation were largely tissue-general, with correlations in methylation between red blood cells (RBCs) and liver [21]. Another study in *P. major* found that methylation patterns from 79% of CGIs were largely conserved between blood and brain tissue [31]. Alternatively, differential methylation of olfactory receptor genes in blood may have direct functional consequences; in humans, olfactory receptor (OR) genes are expressed in nucleated erythroid precursors, presumably owing to the chromatin context of the *β*-globin locus, but this does not indicate functional expression in circulating RBCs [66, 67]. By contrast, white blood cells exhibit ectopic OR transcripts [68], and platelets demonstrate a functional role for OR2L13 [69]. Considered together, the results from the present study and the comparative study of *Catharus* suggest that olfaction may be an important avenue for future work on mechanisms underlying orientation in songbirds.

The patterns of differential methylation we documented here could derive from genetic or environmental variation. Our association analysis suggests they derive at least in part from genetic variants, specifically *trans*-regulatory variants, as a large number of DMSs were associated with *trans* variants, including one of the olfactory genes, which was connected with six SNPs located on chromosomes 1, 2, 4 and 11. Associations with genetic variation are consistent with results from other natural systems. For example, Sepers *et al*. [17] used a partial cross-foster design in a wild great tit population to show that most DNA methylation variation during early development is genetically controlled, allowing for little environmentally induced methylation independent of genotype. Merondun and Wolf [70] also found that DNA methylation was explained in large part by genetic variation in a hybrid zone between crows, although in their case, *cis*-regulatory variants played a larger role. This difference may be related to study design. Merondun and Wolf did not connect differences in methylation with any specific trait; they simply characterized differences in methylation between crows held in a common garden. *Cis*-regulatory variants are thought to underlie species-level differences like this. In our case, we connected differential methylation with a behavioural trait that exhibits intraspecific variation. *Trans*-regulatory variants are thought to be more important at intraspecific levels, especially when it comes to behavioural traits, because they tend to respond more readily to environmental cues. Our finding that *trans*-regulatory variants dominated differential methylation also falls in line with previous work in thrushes, where differences in gene expression were controlled by *trans*-regulatory mechanisms, not *cis*-regulatory changes [45]. Studies on other songbirds also provide evidence for the presence of *trans*-regulatory mechanisms in genetic features associated with migration. For example, researchers who studied zebra finch subspecies found that *trans*-regulatory alterations were more common than *cis*-regulatory changes. These changes were linked to the development and transmission of synapses in song nucleus RA, which is the bird equivalent of the human laryngeal motor cortex [71]. Common garden experiments would be one avenue for future work, not only helping to distinguish between the effects of genetic and environmental variation on differential methylation in the system but also helping eliminate additional factors that could have driven some of the patterns we documented here (e.g. parental effects and/or cultural transmission).

We documented an interesting relationship between methylome-wide variation, heterozygosity and TEs. Specifically, hybrids were isolated from pure forms along the second axis of our whole-methylome RDA, heterozygosity loaded strongly on this axis and many DMSs were in TEs. It has been suggested that during allopatric phases of speciation, incipient species may evolve different mechanisms of repressing TEs that become disrupted (or derepressed) in hybrids when they come back into secondary contact [72]. For instance, a study by Senerchia *et al*. [73] demonstrated that the merging of increasingly divergent long terminal repeat retrotransposons (LTR-RTs), a class of TE, was associated with rising proportions of genome changes in F1 hybrids and involved repeated deletion and methylation of specific LTR-RT loci. TEs can have strong deleterious effects on fitness [74, 75] and thus their de-repression could reduce hybrid fitness, helping maintain species boundaries. Strong reproductive isolation has been documented between Swainson’s thrushes [33, 38, 76, 77]. Differences in migration are thought to generate this isolation, but we may have uncovered preliminary evidence that differential selection on TE regulation and de-repression in hybrids (also termed genomic shock) is also playing a role in maintaining intraspecific variation in migratory direction.

Epigenetic mechanisms are strong candidates for controlling environmentally mediated behaviours like migration. This work is becoming increasingly important with rapidly changing environmental conditions and dramatic declines in songbirds, especially those that migrate [6, 7]. Our results lay the ground for future studies on these topics, using not only larger sample sizes and additional epigenetic markers but also different tissues. Future work could also investigate our preliminary findings for genome shock in the hybrid zone, an exciting avenue by which differences in epigenetic variation could reduce hybrid fitness and help maintain (sub)species boundaries.

## Supplementary Material

supp

Supplementary material is available online at https://doi.org/10.6084/m9.figshare.c.8531181.

Supplementary material is available online [78].
