# Supplementary material for: Epigenetic signatures of migratory orientation in a songbird hybrid zone
Source: Philos Trans R Soc Lond B Biol Sci. Author manuscript; Available in PMC 2026 Aug 18. (PMC13482800; doi:10.1098/rstb.2025.0021)
Supplement: Supplementary file 1 [file NIHMS2195025-supplement-supp.docx]

**Supplementary Table 1.** Samples used in the present study, including average read depth from methylation data, ancestry and heterozygosity estimated from genomic data, fall bearing and classes/orientations used in formal analyses.

| reference | read_depth | ancestry | heterozygosity | fall_bearing | class | orientation |
| --- | --- | --- | --- | --- | --- | --- |
| DH28H02 | 10.04 | 0.94 | 0.08 | 189.19 | coastal | west |
| DH28H14 | 7.56 | 0.93 | 0.11 | 189.19 | coastal | west |
| BH29H09 | 6.97 | 0.91 | 0.17 | 170.58 | coastal | west |
| DI01H10 | 6.78 | 0.97 | 0.06 | 212.49 | coastal | west |
| DH27H03 | 5.59 | 0.97 | 0.05 | 189.19 | coastal | west |
| DH31H11 | 4.99 | 0.94 | 0.10 | 189.19 | coastal | west |
| DH31H06 | 4.52 | 0.78 | 0.32 | 190.52 | coastal | west |
| DI03H03 | 2.87 | 0.95 | 0.09 | 186.85 | coastal | west |
| CH29H09 | 13.57 | 0.42 | 0.60 | 193.33 | hybrid_west | west |
| DH27H12 | 9.51 | 0.46 | 0.62 | 161.32 | hybrid_west | west |
| DH29H08 | 9.19 | 0.49 | 0.58 | 176.69 | hybrid_west | west |
| BH31H04 | 8.84 | 0.46 | 0.90 | 191.66 | hybrid_west | west |
| BI04H06 | 8.54 | 0.60 | 0.72 | 174.26 | hybrid_west | west |
| AH22K02 | 8.48 | 0.51 | 0.73 | 176.48 | hybrid_west | west |
| DI04H03 | 8.13 | 0.53 | 0.70 | 189.19 | hybrid_west | west |
| AH31H04 | 8.12 | 0.43 | 0.69 | 174.30 | hybrid_west | west |
| CI04H07 | 7.82 | 0.55 | 0.68 | 176.69 | hybrid_west | west |
| DH27H06 | 5.86 | 0.56 | 0.68 | 179.63 | hybrid_west | west |
| CH31H03 | 15.80 | 0.43 | 0.71 | 117.20 | hybrid_east | east |
| BI03H06 | 12.41 | 0.39 | 0.56 | 124.52 | hybrid_east | east |
| BH31H08 | 11.34 | 0.47 | 0.63 | 84.09 | hybrid_east | east |
| CH29H07 | 10.68 | 0.53 | 0.44 | 70.52 | hybrid_east | east |
| DH28H04 | 10.14 | 0.48 | 0.46 | 75.86 | hybrid_east | east |
| DI01H12 | 9.81 | 0.46 | 0.53 | 124.49 | hybrid_east | east |
| DH28H05 | 9.53 | 0.61 | 0.53 | 114.37 | hybrid_east | east |
| CI04H08 | 9.00 | 0.50 | 0.61 | 76.72 | hybrid_east | east |
| BI02H08 | 8.13 | 0.43 | 0.68 | 104.88 | hybrid_east | east |
| DH27H10 | 7.39 | 0.44 | 0.67 | 95.86 | hybrid_east | east |
| CI05H01 | 18.39 | 0.09 | 0.13 | 71.79 | inland | east |
| DI02H09 | 17.39 | 0.08 | 0.10 | 84.08 | inland | east |
| CH30H02 | 11.60 | 0.11 | 0.14 | 84.08 | inland | east |
| CH28H04 | 10.86 | 0.10 | 0.13 | 84.08 | inland | east |
| DH30H09 | 8.15 | 0.01 | 0.03 | 70.52 | inland | east |
| DI01H07 | 6.03 | 0.03 | 0.05 | 84.08 | inland | east |
| DH27H13 | 4.48 | 0.08 | 0.10 | 84.08 | inland | east |
| CI09H07 | 4.25 | 0.17 | 0.23 | 84.08 | inland | east |
| DH28H16 | 3.25 | 0.09 | 0.13 | 84.08 | inland | east |
| DH28H17 | 2.18 | 0.05 | 0.09 | 84.08 | inland | east |

**Supplementary Table 2:** Results from GO analysis using genes with DMS between orientations. MF = molecular function. BP = biological process.

| GO ID | term | adjusted p-value | gene number | gene number in pathway | GO category |
| --- | --- | --- | --- | --- | --- |
| GO:0004984 | olfactory receptor activity | 1.58E-06 | 5 | 42 | MF |
| GO:0050911 | detection of chemical stimulus involved in sensory perception of smell | 1.58E-06 | 5 | 42 | BP |
| GO:0007608 | sensory perception of smell | 1.78E-06 | 5 | 47 | BP |
| GO:0050907 | detection of chemical stimulus involved in sensory perception | 7.10E-06 | 5 | 67 | BP |
| GO:0009593 | detection of chemical stimulus | 1.40E-05 | 5 | 81 | BP |
| GO:0007606 | sensory perception of chemical stimulus | 1.57E-05 | 5 | 88 | BP |
| GO:0050906 | detection of stimulus involved in sensory perception | 1.57E-05 | 5 | 89 | BP |
| GO:0051606 | detection of stimulus | 1.22E-04 | 5 | 140 | BP |
| GO:0007600 | sensory perception | 0.0075 | 5 | 337 | BP |
|  |  |  |  |  |  |

**Supplementary Figure 1.** Percentage Methylated Cs in CpG context after removal of C/T and G/A of genetic variant SNP sites. Differences in percentage CpG methylation within and between bird groups were not significant (p = 0.391).
